# Portrait of training and worker health surveillance actions in Santa Catarina

**DOI:** 10.47626/1679-4435-2023-1071

**Published:** 2024-09-24

**Authors:** Sheila Fernanda Madeira, Taís Sparremberger Justo, Renan Ceretta, Flavio Ricardo Magajawski, Willians Cassiano Longen

**Affiliations:** 1 Psicologia, PPGSCol, Universidade do Extremo Sul Catarinense (UNESC), Criciúma, SC, Brazil; 2 Fisioterapia, UNESC, Criciúma, SC, Brazil; 3 Odontologia, UNESC, Criciúma, SC, Brazil; 4 Medicina, Universidade do Sul de Santa Catarina, Tubarão, SC, Brazil; 5 Fisioterapia, PPGSCol, UNESC, Criciúma, SC, Brazil

**Keywords:** worker’s health, occupational health surveillance, health surveillance, saúde do trabalhador, vigilância em saúde do trabalhador, vigilância sanitária

## Abstract

**Introduction:**

Since 2005, the Centro de Referência em Saúde do Trabalhador in the state of Santa Catarina has promoted several training in Vigilância em Saúde do Trabalhador for public health inspectors in the state, so that they can intervene in potentially sick work environments.

**Objectives:**

To evaluate the Vigilância em Saúde do Trabalhador actions carried out by the health surveillance agencies of the state of Santa Catarina and to verify the perception of inspectors regarding the technical preparation for the performance.

**Methods:**

Sample: 257 inspectors from 134 municipalities. Data collection: self-administered questionnaire sent to 733 state inspectors; the number of Vigilância em Saúde do Trabalhador actions planned and executed in 2018, extracted from the Pharos System. The study was cross-sectional, descriptive and with a convenience sample.

**Results:**

Most municipal workers’ health surveillance did not carry out five Vigilância em Saúde do Trabalhador actions per month; most inspectors have difficulties in carrying out Vigilância em Saúde do Trabalhador actions and that they are related to organizational issues. There was no association between the variables training received and actions performed.

**Conclusions:**

It is inferred that the difficulties faced by inspectors may have contributed to the low number of Vigilância em Saúde do Trabalhador actions carried out. It is necessary to make structural and organizational advances in workers’ health in the municipalities.

## Abstract (pt)

**Introdução:**

O Centro de Referência em Saúde do Trabalhador do estado de Santa Catarina promove, desde 2005, diversas capacitações em Vigilância em Saúde do Trabalhador para os fiscais sanitaristas do estado, para que eles possam intervir nos ambientes de trabalho potencialmente adoecedores.

**Objetivos:**

Avaliar as ações de Vigilância em Saúde do Trabalhador realizadas pelas Vigilâncias Sanitárias do estado de Santa Catarina e verificar a percepção dos fiscais a respeito do preparo técnico para a atuação.

**Métodos:**

A amostra foi composta por 257 fiscais de 134 municípios. A coleta de dados foi feita por meio de questionário autoaplicável enviado aos 733 fiscais do Estado e do número de ações de Vigilância em Saúde do Trabalhador planejadas e executadas no ano de 2018, extraídas do Sistema Pharos. O estudo foi transversal, descritivo e com amostra de conveniência.

**Resultados:**

A maioria das vigilâncias em saúde do trabalhador municipais não realizaram cinco ações de Vigilância em Saúde do Trabalhador por mês; a maioria dos fiscais têm dificuldades na execução das ações de Vigilância em Saúde do Trabalhador e que elas se relacionam às questões organizacionais. Não foi verificada associação das variáveis capacitações recebidas e ações realizadas.

**Conclusões:**

Infere-se que as dificuldades enfrentadas pelos fiscais possam ter contribuído para o baixo número de ações de Vigilância em Saúde do Trabalhador realizadas. É necessário que se façam avanços estruturais e organizacionais da saúde do trabalhador nos municípios.

## INTRODUCTION

Work plays an important role in the health-disease process. In 2020, the Anuário Estatístico da Previdência Social (AEPS – Social Security Statistical Yearbook) recorded 445,814 work-related accidents.^1^ However, there are still millions of workers not included in this number, such as civil servants, military personnel, self-employed workers, cooperative workers, and service providers, to name a few.^2^

When turning the numbers into figures, the Observatório Digital de Saúde e Segurança no Trabalho (Digital Observatory of Occupational Safety and Health) of the Ministério Público do Trabalho (Public Prosecutor’s Office) showed that, between 2012 and June 2019, more than R$84 billion were spent on accident benefits.^3^

This scenario of high occupational accident rates is not unique to the 2000s. It first appeared on the scene in 1970, when Brazil was ranked first in the world for occupational accidents.^2,3^ It is therefore correct to say that there is a public health problem that is addressed in an inexpressive or insufficient manner, given the dramatic magnitude of these indicators.^2^

However, this is not due to a lack of legal provisions, as occupational health has been included in public health since 1988, with the Constituição da República Federativa do Brasil (Constitution of the Federative Republic of Brazil), which states in article 200 that “the Sistema Único de Saúde (SUS, Unified Health System) is responsible for (...) executing occupational health actions (...)”, and also for “(...) collaborating to protect the environment, including the work environment....”^4^ The Lei Orgânica da Saúde (Organic Law on Health) came into force two years later, stating:

For the purposes of this law, occupational health is understood to be a set of activities aimed, through epidemiological and health surveillance actions, at promoting and protecting workers’ health, as well as recovering and rehabilitating the health of workers subjected to hazards and harms arising from working conditions....^5^

In 2002, in an effort to systematize and organize occupational health actions, the Rede Nacional de Atenção Integral à Saúde do Trabalhador (RENAST, Brazil National Network for Comprehensive Occupational Health Care) and the Centro de Referência em Saúde do Trabalhador (CEREST, Brazil Occupational Health Reference Centers)^6^ were created.

The weaknesses of this network include the unpreparedness of professionals working in the service network to deal with work-related health risks and hazards, the precedence of care over surveillance actions and the timid approach to intersectoral actions.^6^

In 2012, the Política Nacional de Saúde do Trabalhador e da Trabalhadora (PNSTT, Brazil National Occupational Health Policy) was instituted, and its article 9 highlights the need for “investments in the integrated qualification and training of teams of various components of health surveillance (...).”^7^

Although the Vigilância em Saúde do Trabalhador (VISAT, Brazil Occupational Health Surveillance) was created to promote and protect workers’ health, practice has shown that the lack of professionals with a degree and qualifications, among other factors, hampers the effectiveness of this surveillance.^8^

CEREST, fulfilling its role of structuring and coordinating RENAST, has promoted training in occupational health for health inspectors from all municipalities in Santa Catarina, to provide technical support for their work in the workplace since 2005. Thus, local health surveillance agencies (VISAs) could be partly responsible for implementing actions through agreements, in a safe and effective manner.^9^

Although we recognize the importance of this initiative, no evaluation of implemented VISAT actions have been found, whether they correspond to the number of actions agreed, or whether the training offered, as perceived by inspectors, is qualifying them technically for effective VISAT actions.

This study therefore aimed to evaluate the VISAs VISAT actions taken in Santa Catarina, and to ascertain the inspectors’ perception of their technical qualifications for their effective action.

## METHODS

This study followed ethical principles and was approved ethically and methodologically by the Human Research Ethics Committee (CEP), in accordance with international and Brazilian guidelines and standards, under protocol No. 3.889.200.

This was a descriptive, cross-sectional study with a convenience sample, conducted in the VISAs of 134 municipalities in Santa Catarina. The population included 733 health inspectors, according to a list provided by the Diretoria de Vigilância Sanitária de Santa Catarina (DIVS-SC, Health Surveillance Directorate in Santa Catarina). The convenience sample totaled 257 inspectors, distributed across 134 municipalities in Santa Catarina. The sample calculation showed that 253 questionnaires would have to be answered for a 95% confidence interval and a 5% margin of error.

The data was collected using three instruments: 1) the Pharos System, which is an online tool that acts as a single, integrated database in Santa Catarina, accessible and fed by all local VISA units, regional health units, and the DIVS-SC; 2) a structured, self-administered questionnaire to assess the inspectors’ perception of their technical preparedness to work, and other variables that may or may not be involved in performing VISAT actions.

On April 1, 2020, data collection began with sending an informed consent form (ICF) and a Google Forms’ questionnaire to a DIVS-SC list of emails of 733 health inspectors; the questionnaires were sent twice to those who had not completed them. In total, 265 questionnaires were answered. We excluded eight questionnaires that were left blank. As a result, we reached the number of questionnaires that comprised our sample.

After the questionnaires were completed, the Pharos system was accessed to collect data on VISAT actions planned and executed in 2018 in 134 municipalities in Santa Catarina.

The data was tabulated and analyzed using the IBM Statistical Package for Social Sciences, version 20.0 (SPSS, Inc., Chicago, USA). Categorical variables were expressed as absolute and relative frequencies. Numerical variables were expressed as mean, standard deviation (SD) and median.

Pearson’s correlation coefficient was used to verify the association between the categorical variables (the number of VISAT actions planned and the number of actions executed; the number of training courses attended and the number of actions executed), with a significance level of 5%.

## RESULTS AND DISCUSSION

The mean age of the health inspectors who participated in this survey was 40.9 years, and most were women (59.1%).

As for their length of service as inspectors, the median was 5 years, ranging from 1 to 35 years. Thus, the mode was the same, with 31 inspectors working for 5 years.

Considering the mode, the number of VISA inspectors most often found is one (52 responses) or two (47 responses). This means that 99 inspectors answered that there are only one or two health inspectors in their municipality, which is 38.5%.

This figure gains even more relevance when you realize that 210 inspectors (81.7%) responded that it is difficult to execute VISAT actions. Of these, 134 reported that the demand exceeds the number of inspectors and 144 said that it is a challenge to take action in different areas, an issue further discussed in Table 1.

**Table 1:** Data distribution on the execution of VISAT actions

Execution of VISAT actions	n (%) n = 257
Actions taken	
Not implemented	67 (26.1)
Planned routine actions	23 (8.9)
Actions on demand from external bodies	84 (32.7)
Routine actions and on demand	50 (19.5)
Other actions	17 (6.6)
Challenges	
Yes	210 (81.7)
No	47 (18.3)
Main challenges	
No staff training	80 (31.1)
Few training courses	93 (36.2)
Few inspectors to meet demands	134 (52.1)
Execute actions in different areas	144 (56.0)
Interference from politicians	65 (25.3)
Productivity goals	12 (4.7)
Support from managers	52 (20.2)
Poor infrastructure	40 (15.6)
Other	9 (3.5)

VISAT = Vigilância em Saúde do Trabalhador. (Brazil Occupational Health Surveillance)

As for the execution of activities in different areas, Silva et al.^10^ stressed that the VISA covers food; medicines; medical, hospital, dental, and laboratory products; sanitizing, health, and health-related services; sanitary control of tobacco products, to name a few. This finding is consistent with the response of most inspectors, who believe that their work in several areas presents a challenge to the full implementation of surveillance measures.

Table 2 shows the data collected on occupational health training and inspectors’ participation in these training courses.

**Table 2:** Occupational health training courses

Training courses	Median (min.-max.), n (%) n = 257
Participated in occupational health training courses	
Yes	171 (66.5)
No	86 (33.5)
Number of training courses	1.0 (1.0-20.0)
Training courses attended	
Basic VISAT actions	152 (59.1)
Basic inspection actions at service stations	35 (13.6)
Analysis of safety and health documents	18 (7.0)
Regulatory standards	36 (14.0)
Ergonomics	15 (5.8)
Other	23 (8.9)
Need for further training courses	
No need for further training courses	3 (1.2)
Basic VISAT actions	66 (25.7)
Basic inspection actions at service stations	42 (16.3)
Analysis of safety and health documents	58 (22.6)
Regulatory standards	62 (24.1)
Ergonomics	44 (17.1)
Other	0 (0.0)

VISAT = Vigilância em Saúde do Trabalhador (Brazil Occupational Health Surveillance).

Most inspectors (66.3%) have already attended at least one training course on occupational health. Over the last 15 years, Santa Catarina CEREST has organized several training courses from 2005 to 2009, including one which included occupational health as part of the basic content of the training course for state health inspectors. Remarkably, 59.1% of inspectors have attended this course.

A study conducted in Itaberaba, Bahia, found that local health inspectors began to incorporate occupational health into their daily work routine through a gradual and continuous process of training.^11^

Of the 171 inspectors who reported having already attended some training, 100 had only attended one. Considering the 38 inspectors who had only attended two, we have 138, which is a modest number, in view of the complexity of current work processes and environments.

Limongi et al.^8^ found, when studying the structure and processes of health surveillance in municipalities in Minas Gerais, that one of the factors hindering the progress of VISAT actions is the need for professionals trained in occupational health. Given the importance of this training, we cannot ignore the fact that 33.6% of the inspectors had not been trained, although it was not the majority of responses.

Table 2 shows that 33.5%, nearly one third of the sample, have not attended any occupational health training courses. This finding raises a number of questions about the reasons why inspectors may not participate, even when they are offered the opportunity: the distance from many municipalities to Florianópolis; inspectors from municipalities with only one inspector may find it difficult to leave VISA for several days; permission denied from their managers; no funds for transportation and food; etc. A plausible alternative to increase attendance is distance learning, which was widely available during the COVID-19 pandemic.

All the alternatives regarding the need for more training topics were ticked, especially Basic VISAT actions, Regulatory standards, and Analysis of safety and health documents, respectively 25.7%, 24.1%, and 22.6% of responses. This result is consistent with our findings because this study assessed the effectiveness and satisfaction of training for VISA technical staff in Uberlândia, Minas Gerais. Most participants in this mini-course suggested that further training be given on various important and necessary subjects. The scarcity of training for inspectors in Uberlândia was noted, which directly interferes with inspections.^12^

Table 3 shows the results for the quality of the training courses the inspectors attended. Most inspectors considered that the training courses partly provided sufficient and appropriate technical support to execute VISAT actions, 61.4% and 59.1% respectively. Those who answered “yes” to both questions were 24.6% and 28.1%. Table 3 shows the data on the execution of VISAT actions.

**Table 3:** Distribution of data on the quality of occupational health training

Occupational health training courses	n (%) n = 171
Provided sufficient support for execution	
Partly	105 (61.4)
Yes	42 (24.6)
No	24 (14.0)
Provided adequate support for execution	
Partly	101 (59.1)
Yes	48 (28.1)
No	22 (12.9)
The content was clear	
Partly	45 (26.3)
Yes	117 (68.4)
No	9 (5.3)
The learning methodology was appropriate	
Partly	50 (29.2)
Yes	108 (42.0)
No	13 (5.1)

When asked about how VISAT actions are taken, 32.7% of inspectors stated that they are executed when they are demanded by external bodies. Importantly, these are not joint actions involving several agencies, but rather those who demand the action.

Chaves & Seta^13^ stated differently in their study on VISAT at floating service stations in Manaus, AM. They highlighted the importance of VISA in joint actions with other bodies, such as CERESTs, trade unions, and universities, as health inspectors have enforcement powers which, combined with the specificities of other bodies, can effectively transform workplaces. Similarly, Seta et al.^14^ found that VISAs need strong intersectoral coordination to ensure their health surveillance actions are effective.

We should also highlight that 26.1% do not execute VISAT actions. Chaves & Seta^13^ reiterate the importance of local health surveillance in inspecting workplaces, processes, and conditions, since it is the competent health authority for inspecting all establishments under its jurisdiction.

The SUS Instrução Normativa (IN, Normative Ruling) on VISAT, which is currently included in Annex LXXIX of the Portaria de Consolidação (Consolidation Ordinance) No. 1, of 28 September 2017,^15^ has contributed structurally to the organization of surveillance and other actions in occupational health services in the various administration levels of the SUS. In relation to VISA actions, this IN reaffirms sanitary inspection in occupational health. It also provides guidelines on the need to comply with the rules and legislation that regulate occupational health relations, from any source, especially within health, labor, social security, environment, and international agreements and conventions Brazil has ratified. This IN also highlights the relevance of aspects that could cause harm to health through direct observation or through issues referred to from administrative instruments already used by the Health Surveillance/Inspection, such as the Termos de Visita (Terms of Visit), Notificação (Notification), Intimação (Intimation), Auto de Infração (Notice of Infraction), and other instruments.

The document which sets out the guidelines on how to implement VISAT in the SUS reaffirms the need for surveillance professionals to be assigned by health authorities so that inspections of workplaces can be conducted.^16^

However, contrary to the consolidation of occupational health actions in the SUS, surveillance actions are timid, since CERESTs hold no health authority, hindering direct intervention in production processes and workplaces.^17^ This survey results reiterate the scarcity of planning in occupational health, only 8.9% routinely execute planned actions.

VISAT actions, despite the creation of RENAST, are still residual within the SUS, and progress has occurred more from a conceptual point of view than a practical one. Despite the various experiences, there are few cases that can be considered as executing systematic actions, and in several states and municipalities they do not exist at all.^18,19^

The scarcity of more ostentatious, planned, and routine VISAT initiatives contributes to a failure to reduce accidents, diseases, and deaths at work.^20^ Recktenwaldt & Junges^21^ corroborated the lack of planning in surveillance actions, including VISAT, as they found no planning in three of four municipalities in Rio Grande do Sul.

Table 1 shows an important point for reflection: 81.7% of inspectors find it difficult to implement VISAT actions.

Penariol & Benelli^22^ reported on the difficulties VISAs encounter in the execution of occupational health actions in an inland, medium-sized municipality located in the São Paulo. These challenges even prevented effective intervention, and the following issues stand out: VISA staff demotivation; no clear objectives for their work; poor training in occupational health; extremely limited access to the internet; poor or inexistent networking in the municipality; no political interest in implementing the PNSΊT; failure to inspect and/or notify; and bans on inspections of certain sites due to impediments caused by interests of political groups.

As for the main obstacles the inspectors faced in the survey, 56% reported having to take action in different areas and 52.1% stated that VISAs have too few inspectors to cope with demands. Both responses are complimentary because, theoretically, if more inspectors were available, it would be also possible to assign an exclusive team to VISA sub-areas, including occupational health.

The main challenges this survey revealed were few or no training courses; 36.2% and 31.1% of the responses, respectively. The inspectors mentioned this as a problem, even when training was provided by their state CEREST. This finding allows for a discussion about the difference between specific training and Permanent Health Education (PHE). Both certainly play an important role in the development of public agents, but the results in everyday life tend to be different.

The PNSΊT emphasizes the need for training through PHE, complying with the guidelines of the Política Nacional de Educação Permanente em Saúde (PNEPS, Brazil National Policy for Permanent Health Education), and encourages partnerships between relevant bodies and institutions to provide training and capacity building for the community, workers, and social control.^7^

When the characteristics of PHE are considered, it is practically impossible for a state body like CEREST to implement PHE on occupational health in all Santa Catarina municipalities. However, training courses should, among other things, act as a “trigger” for local CERESTs, health departments, and health secretariats to continue the process:

XIII - to train SUS health professionals and teams, in partnership with the Secretarias Estaduais de Saúde (State Health Secretariats) and CERESTs, to identify and act in situations of work-related health risks, and to diagnose work-related hazards, consistent with the guidelines for implementing the Política Nacional de Educação Permanente em Saúde (Brazil National Policy for Permanent Health Education) [...].^7^

Article 14(I) of the PNSTT^7^ reiterates the role of the local CERESTs in providing technical support, continuing education, coordination of projects to promote and monitor occupational health.

Two recent studies corroborate the prominent role CERESTs play in this methodology, demonstrating that matrix support has enabled more effective interventions. They also showed the potential of the method to improve communication between specialized services and those at the “front line,” helping to solve problems and guide occupational health actions, including health surveillance and health promotion.^23,24^ Although in these two cases the matrix support involved professionals from primary care and local CERESTs, their important role in training and PHE should not be overlooked.

The correlations between the variables used in this study are shown in the following two tables (Tables 4 and 5).

**Table 4:** Correlations between planned VISAT actions vs. executed actions

	n	r_s_*	p-value
Chapecó	33	0.286	0.148
Criciúma	20	0.319	0.196
Itajaí	13	0.244	0.527
Mafra	7	0.674	0.142

VISAT = Vigilância em Saúde do Trabalhador (Occupational Health Surveillance).

*Spearman’s correlation coefficient.

**Table 5:** Association between training courses attended and executed actions

Training courses attended	Actions taken, n (%)					p-value*
	0-2 n = 70	3-5 n = 10	6-8 n = 2	9-12 n = 5	≥ 13 n = 22	
0-2	54 (67.5)	9 (11.3)	2 (2.5)	2 (2.5)	13 (16.3)	0.022
3-5	12 (63.2)	1 (5.3)	-	2 (10.5)	4 (21.1)	
6-8	2 (40.0)	-	-	1 (20.0)	2 (40.0)	
9-12	-	-	-	-	2 (100.0)†	
**≥** 13	2 (66.7)	-	-	-	1 (33.3)	

*Value obtained after the linear-by-linear association test has been applied.

^†^Statistically significant value after residual analysis.

Table 4 groups the municipalities into regions. It shows the correlation between planned VISAT actions and executed actions. In none of them was the result statistically significant (p < 0.05), i.e. there was no relationship between the number of executed actions and planned actions. The correlation test could not be performed for the other regions because the variables relating to planned actions were constant.

Table 5 shows an association between the number of training courses attended versus the number of actions executed per region.

Overall, no association was found between training courses attended and executed actions. The only range that contributed to a 0.022 p-value was that of 9 to 12 training courses attended with 13 or more executed actions, i.e. only in this range significant association between these two variables was found. Thus, the more VISAT training attended, the more executed VISAT actions.

The results of the last two tables provide food for thought and discussion as to whether providing training on topics relevant to the everyday work of inspectors is not enough to make VISAT actions a reality.

It is therefore worth reiterating some of the results and issues already covered in this discussion which, at this point, can help us understand the possible factors that contributed to no association among the variables shown in Tables 4 and 5.

When we look back at the data in Table 1, 81.7% of the inspectors reported facing problems when working on surveillance actions, the two most frequently mentioned were having to take actions in different areas and having too few inspectors for all of the VISA demands. These two organizational aspects can interfere negatively in the execution of VISAT actions because, in addition to the insufficient number of inspectors, they also need to execute actions in different areas, which are part of the scope of VISAs, although they can be organized by each municipal service.

Similarly, a study conducted with surveillance agencies in small municipalities in Rio Grande do Sul found that professionals pointed to the overlapping of duties, which was justified due to a lack of financial resources to respond to the new surveillance duties resulting from a decentralization process. Professionals consider this overlapping of duties to devalue health surveillance agencies, which do not have the proper conditions to fulfill their duties and tasks in their region.^21^

VISAT actions can also be hampered if they are not planned. The strategic planning process proposed by the SUS presents responsibilities that should be continuously configured, articulated, integrated, and mutually shared between the three federated levels, so as to favor the implementation of actions through commitments and help develop effective methods and strategies to obtain satisfactory results.^25^

This study has some limitations, notably its cross-sectional design, in which the variables were obtained at a specific time interval, thus not allowing a causal relationship to be established; missing information in the Pharos System for several municipalities; and the self-administered questionnaires, sent over the internet, which essentially have limitations such as impersonality.

## CONCLUSIONS

The results of this study and other studies have shown that the difficulties faced by inspectors in their working routine, such as insufficient human resources, actions being executed in various sub-areas of health surveillance by the same inspector, poor planning and intersectoral coordination may have contributed to training courses not having a direct impact on the number of VISAT actions.
